# The Size Screening Could Greatly Degrade the Health Risk of Fish Consuming Associated to Metals Pollution—An Investigation of Angling Fish in Guangzhou, China

**DOI:** 10.3390/toxics11010054

**Published:** 2023-01-04

**Authors:** Xiongyi Miao, Qian Zhang, Yupei Hao, Hucai Zhang

**Affiliations:** 1 School of Geography and Environmental Science & School of Karst Science, Guizhou Normal University, Guiyang 550001, China; 2 Key Laboratory of Karst Dynamics, MNR&GZAR, Institute of Karst Geology, CAGS, Guilin 541004, China; 3 Department of CPC Organization and Human Resource, The First Affiliated Hospital of Guangxi Medical University, Naning 530021, China; 4 Institute for Ecological Research and Pollution Control of Plateau Lakes, School of Ecology and Environmental Science, Yunnan University, Kunming 650500, China

**Keywords:** angling fish, metals contamination, health risk, size screen, Guangzhou City

## Abstract

Fish size can heavily impact the bioaccumulation of metals, but it was rarely applied to screen out the fish with low health risk for consuming. Given the widespread metals contamination of angling fish, the angling fish collected from Guangzhou, China, were taken as an example in this study. The screening length and weight were detailed in accordance with the investigation of metals contamination among angling fish. Importantly, the feasibility of size screening on mitigating the health risk of angling fish was evaluated. The results revealed that the concentration of Cr and As were relatively high and beyond the maximum residue limit (MRL) in some fish. The mean pollution index (Pi) of As, Cr, and Pb were beyond 0.2, suggesting the widespread minor contamination. The total metal pollution index (MPI) manifested *Oreochroms mossambcus* was the most contaminated. The target hazard quotient (THQ) of Cr, As, and Hg were relatively higher, but the higher probability of THQ > 1 indicated the health risk should be dominantly from As. The highest TTHQ suggested the highest risk of *Oreochroms mossambcus*. Regression analysis determined the fish of THQ < 1 should be more likely centralized in the size that is beyond 13.7 cm and 45.0 g for adults and 19.8 cm and 127.9 g for children. Significantly reducing THQ among these screened fish confirmed their effect on the degrading health risk of metals; particularly, the children’s THQ returned below 1. The commonly contaminated *Oreochroms mossambcus* was further excluded to remove the screened fish with THQ > 1; the further decrease in THQ confirmed that the exclusion of a contaminated species could improve the effect of size screening.

## 1. Introduction

Fish are rich in unsaturated fatty acid (DHA and taurine), trace elements, and protein. Their digestibility is confirmed to be significantly higher than that of chicken, lamb, beef, and pork, so that fish are generally considered to be the most important source of various nutrients [1,2]. Despite the well-being of fish consuming being widely confirmed in decreasing the risk of various diseases and promoting the normal development of the central nervous and visual systems and the fetal brain [3], it does not mean that fish consuming cannot harm human health. On the contrary, the health risk of fish consuming also hides beneath the well-being of fish consuming, which dominantly resulted from the excessive uptake of various contaminants from the consumption of the contaminated fish, particularly metals contamination [2,4]. Compared to market fish that is under severe surveillance, wild fish should be more susceptible to metals contamination [5]. However, given the invisibility of metals contamination and no additives during growth, wild fish, instead of market fish, were advertised to be more healthful for consuming, so that wild fish were highly sought, which also made wild fish become a critical source of metals for human [2,6].

In the context of the sharp decline in biodiversity and the depletion of fishery resources, fishing moratoriums and bans have turned into the universal practice for the restoring of rivers [2,7,8]. However, the recreational fishing was rarely incorporated into the category of fishing bans, so that recreational fishing became the primary way to obtain wild fish [1,8]. More than 220 million to 700 million anglers were reported to be active across the world [9], among them, the active anglers were estimated to be more than 90 million in China [1]. Despite the widespread contamination of metals among angling fish that was disclosed before [2,4,10,11], given the scarcity of wild fish, angling fish were still largely consumed by anglers and their families, which make it very hard to reduce the health risk of angling fish consuming by only relying on persuading the public to refuse angling fish consuming, so that the decreased health risk of angling fish consuming should be still depended on to degrade the contamination of metals among angling fish itself. Therefore, developing a new approach to reduce the metals contamination of angling fish is urgently needed for safer fish consuming. The bioaccumulation of metals among fish was formerly confirmed to tightly relate to the growth of fish [1,12,13,14]. Junior fish are richer in heavy metals than senior fish because of the effect of biological dilution of metals [2,15]. Given the appropriate expression of fish length and weight on fish growth, the size comparison based on length and weight can be used to determine the relative contamination of metals among angling fish [1]. However, the comparison of length and weight would be useless to screen out the fish with a low risk for consuming. In order to guarantee the health risk of fish consuming within the acceptable range, it is essential to establish the correlations between fish size and health risks of metals, which is critical to reach the goal of degrading the health risk of angling fish consuming.

Guangzhou City, located in the center of Pearl River Delta in southern China, is one of the most densely populated industrial cities in China. The prosperous industries and high urbanization have made Guangzhou City become an important city with a huge discharge of effluent. The annual emission of Cd, Pb, Cr, Cu, and Zn were reported to be 0.4, 1.7, 43, 705, and 10,304 tons, respectively, most of which were directly into Pearl River with less treatment [16]. This makes metals the main contaminants appearing in Pearl River [17,18,19]. Despite the contamination of metals among wild fish in Pearl River confirmed in previous studies [20,21], it did not subsided the enthusiasm of fish angling. Anglers are still widely distributed on the bank of Pearl River, particularly in urban areas, to obtain the wild fish for consuming as usual. Given the huge risk of contaminated fish consuming, the consumption of angling fish from Guangzhou City should be guide in a more suitable way of consuming, instead of just limiting the consumption in vain. The screening length and weight can differentiate metals bioaccumulation among fish [1], but their feasibility on mitigating the health risk of fish consuming have not been verified, particularly for the angling fish of Guangzhou City, which is not conducive to degrade the health risk of angling fish consuming in Guangzhou City. Additionally, despite some studies reporting metals contamination of wild fish in the whole Pearl River Delta [20,21], the prevalence of contaminated fish and the fish with an unacceptable risk still are unevaluated; given the more dense industries in Guangzhou City, the investigation of metals contamination among wild fish still needs to be conducted in Pearl River of Guangzhou Section, which is conducive to determining the situation of metals contamination in Guangzhou City. For this purpose, the study aimed to (1) investigate the distribution of eight metals (i.e., Cu, Pb, Zn, Cr, Cd, As, Hg, and Se) in angling fish of Guangzhou City, (2) evaluate the prevalence of contaminated fish and the fish with an unacceptable risk in Guangzhou City, and (3) analyze the feasibility of size screening on mitigating the health risk of angling fish consuming. The size screen proposed in this study will not only improve the guidance of angling fish consuming, but also promote the environmental monitoring, control, and governance of the river.

## 2. Materials and Methods

### 2.1. The Description of Study Area and Field Sampling

**Figure 1:**
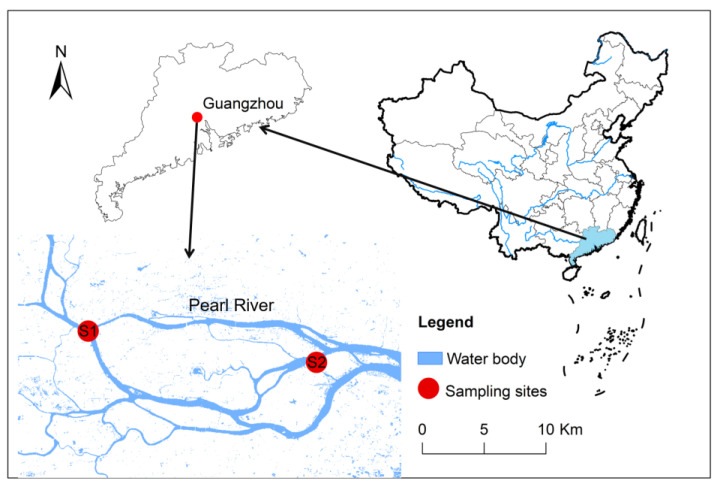
Study area in the Guangzhou section of Pearl River.

Guangzhou City is located in Guangdong Province in China. The most important surface runoff in Guangzhou City is Pearl River [16]. The Guangzhou section of Pearl River, including the western, front, rear, and Huangpu channels, is the main water body in Guangzhou City, the total length being 82.55 km and the water square being 38.9 km^2^ [17]. The daily volume of sewage drainage of Guangzhou is around 200 million tons. More than 70% of sewage is drained to the Pearl River directly without any treatment, which profoundly affected the ecosystem of Guangzhou section in Pearl River [18,19]. Given the angling fish was mainly caught in urban areas, two gathering spots for fish angling in urban areas were selected as the sampling plots in this study (Figure 1). Among them, S1 is located at the upstream urban area in Guangzhou City, which is just along the bank of Shamian Park in Yuxiu district, while S2 is located at the downstream urban area in Guangzhou City, which is along the bank of Zhongshan Park in Changzhou island. The fish samples were directly obtained from the anglers located in every selected angling area, from 10–18 July 2021. Small fish were excluded from this study, as fish below the size of 10 cm and 20 g are rarely eaten by anglers. A total of 137 fish, belonging to 11 species, was collected from S1 and S2. The species, size, and location of these fish were recorded after sampling. All of these fish samples were stored in a self-sealed polythene bag under 20 °C before further processing. The details of the fish samples are shown in Table S1. The higher quantity ratio of *Cyprinus carpio* and *Oreochroms mossambcus* suggested they should be considered to be the dominant species of angling fish in Guangzhou City.

### 2.2. Sample Preparation and Analysis

The fish length, weight, and species were determined and recorded in the laboratory (Table S1). As the most consumed part of fish, only the back muscle of fish was extracted to test, with amounts of 50–100 g. More than three fish with approximate length or weight were combined into one to meet the requirement of minimal test weight. After washing with Milli-Q water, the back muscle of fish was frozen dried for 72 h at −80 °C to constant weight and then powdered to a uniform particle size with the grinder. Powdered fish tissue of 0.3 g (dry weight) was put into a microwave digestion tube and digested with 10 mL of HNO_3_ (68%) and 2.0 mL of H_2_O_2_ (30%) in a microwave digester at 140 °C for 6 h. AFS-920 (AFS-920, Titan Instruments, Shanghai, China) and ICP-MS (ICP-MS, Thermo X series, ThermoFisher, Waltham MA, USA) were, respectively, used to determine the content of Cu, Pb, Zn, Cr, Cd, As, Hg, and Se [2]. The metals contents in wet weight of fish were calculated with the before and after frozen dried weight of the fresh fish flesh. The description of quality assurance and quality control were found in our previous literature [1].

### 2.3. Assessment Method

#### 2.3.1. Assessment of Metal Pollution

The metal pollution index (Pi) was used to evaluate the contamination of metals among angling fish [1,2]. The Pi of Cu, Pb, Cr, Cd, As, and Hg were calculated by the following Equation (1):

Pi = Ci/Csi(1)

In this equation, Pi is the monomial pollution index of metal i; Ci and Csi are, respectively, the content and the threshold values of metal i in fish samples (mg/kg wet weight). The Csi of Pb, Cr, and Cd referred to the maximum residue limits of contaminants in food in China [22], and the Csi of Cu, total As, and total Hg were within the safety requirements for non-environmental pollution of aquatic products in China [23].

The Pi values are divided into four pollution levels, namely no significant pollution (Pi < 0.2), minor pollution (0.2 < Pi < 0.6), moderate pollution (0.6 < Pi < 1), and severe pollution (Pi > 1). The total metal pollution index (MPI) was used to assess the comprehensive metals pollution in fish. The concentration of metal n was represented by Cn, and MPI was calculated by the Equation (2):

MPI = (C1 × C2 × C3 ×……Cn)^1/n^ (2)

#### 2.3.2. Health Risk Assessment of Fish Consumption

The target hazard quotient (THQ) was used to estimate the health risk of those metals that exceeded the limits established by relative legislation [24]. THQ is the ratio between the exposure contaminant and the reference dose (RfD):

(3) $$\mathrm{THQ}=\frac{\mathrm{EF}\times \mathrm{ED}\times \mathrm{IRd}\times C}{\mathrm{RfD}\times \mathrm{BW}\times \mathrm{AT}}$$

The THQ value < 1 suggests that the exposed population is unlikely to suffer obvious adverse effects, while the THQ value > 1 expresses that the level of exposure is beyond the oral reference dose, so that effective interventions and protective measurements should be taken [21]. IRd, EF, ED, BD, AT, and RfD in Equation (3) are the ingestion rate of people (g/day), exposure frequency (days/year), exposure duration (year), body weight (kg), average time of exposure (day/year), and oral reference dose (RfD) (μg/kg/day), the values of which could be found in our previous study [2].

The calculation parameters of children and adults involved in Equation (3) were according to Miao [2]. When calculating the THQ of Hg, the total concentration of Hg in the muscle tissue was assumed to be equal to that of MeHg, which has also been used by previous researchers [2]. The total target hazard quotient (TTHQ) is equal to the sum of each metal THQ value, which can be presented as:

TTHQ = ∑THQ(4)

### 2.4. Statistical Analysis

Excel 2010 was used to perform the data analysis. The tables and figures were done on OriginPro 8 and Coreldraw X7. The analysis of correlation, regression, and Monte Carlo were employed in this study with the help of SPSS 22, OriginPro 8 and Oracl Crystal Ball.

## 3. Results and Discussion

### 3.1. The Metals Concentration of Angling Fish in Guangzhou City

The metals concentration of angling fish is shown in Table 1; the average content of metals in angling fish decreased as follows: Zn > Fe > Mn > Ni > Se > Cu > Cr > As > Pb > Hg > Cd. The maximum and minimum of metals were determined to be Zn and Cd, respectively, the mean of Zn reached to 16.723 mg/kg (wet weight), while the average Cd was only 0.004 mg/kg (wet weight). Comparing with the wild fish in Pearl River Delta, almost all metals in angling fish of this study are significantly lower, which suggested the aquatic environment in Guangzhou City was relatively better that of other cities in Pearl River Delta. Therefore, Guangzhou City should be not treated as the hot spot of the sewage emission in Pearl River Delta. Compared to the rivers in other Chinese drainage areas, Zn, Cr, and As were relatively higher in the angling fish of Guangzhou City, which suggested the more intense emission of these elements in Pearl River Deltas. Comparied to market fish of China, Pb, Cr, and As were significantly higher, which indicated the consumption of these angling fish may aggravate the exposure of Pb, Cr, and As. Despite the mean concentration of metals in angling fish all being below the corresponding MRL of metals in fish recommended by the Chinese government, the content of Cr and As in some angling fish were still found to be exceeding their corresponding MRL, which suggested Cr and As should be critical for manipulating the security of angling fish consuming.

The metals concentration of the fish in different species is given in Figure S1. According to the distribution of the maximal values of each metal, the bioaccumulation of metals was not found to be centralized in the species of a small minority, but well distributed to most species, which suggested the variation of metals should be considered to be significant in different species. *Pelteobagrus fulvidraco* were enriched with the higher content of Cu and Cr, whereas, in *Oreochroms mossambcus*, the higher content of As and Pb were observed. Similarly, *Carassius auratus* contained more Zn, *Ophiocephalus argus Cantor* demonstrated significant accumulation of Hg, *Cirrhinus molitorella* contained abundant quantities of Se, and *Prochilodus scrofa* were rich in Cd. These mentioned fish species are all demersal, which suggests the demersal habitats may aggravate the bioaccumulation of metals in angling fish [27]. For the minimal value of metals, *Aristichthys nobilis* contained less As; the lower content of Zn and Cr were only found in *Clarias fuscus*, *Ctenopharyngodon idellus* accumulated less Cd, while *Pseudohemiculter dispar* were enriched with less content of Cu. Among these four species with low content of metals, half of them were found to be pelagic, i.e., *Aristichthys nobilis* and *Pseudohemiculter dispar*; their lower accumulation of metals suggested that pelagic habitats should play an important role on mitigating the accumulation of metals in angling fish [13]. For the feeding behaviors of fish, the herbivore fish were formerly confirmed to be able to partially impair their bioaccumulation of metals by degrading their trophic levels [28,29], which were also expressed on the overall low content of metals in *Ctenopharyngodon idellus*. Therefore, the bioaccumulation of metals in angling fish should be not only heavily impacted by the living habitats but also influenced by the feeding behavior.

With the exception of living habitats and feeding behavior, the growth of fish can also heavily impact metals bioaccumulation in fish [1,12,13], which is well expressed on the variation of metals bioaccumulation between junior and senior fish. Junior fish are in the period of rapid growth and need massive feeding to sustain growth, but the insufficiently developed organs degrade the metals metabolism of junior fish, so that metals can accumulated more in junior fish [1,30]. However, for the fully developed senior fish, they are already in the period of slow growth and do not need too much feeding, so that they can metabolize most metals, which results in the commonly low metals accumulation in senior fish [1,31]. These variations of metals bioaccumulation from junior to senior fish were treated as the effect of bio-dilution [13,15]. As the direct indicator of fish growth, the correlation of the length and the weight of fish was commonly confirmed to be negative with metals concentration in this study (Table S2), which suggested the bioaccumulation of metals in angling fish of Guangzhou City was totally conformed to the effect of bio-dilution, so that the fish length and weight can be used to differentiate the bioaccumulation of metals in angling fish. In other words, fish length and weight have a high potential for screening the fish with a low risk of metals. Given the overall higher correlation coefficients of fish length, metals should be more likely centralized in fish with small length, so that fish length, instead of fish weight, was more superior to differentiate the bioaccumulation of metals in angling fish. For each metal, the correlation coefficients of Zn, As, and Pb were relatively higher than other metals either with length or weight, which suggested their preference for accumulating in junior fish, so that size screening may have more influence on the health risk of angling fish consuming associated to these metals. The correlation of fish length and weight were found to be non-significant with Hg, which suggested the overall even distribution of Hg in different fish. Given the higher toxicity of Hg [32], the bioaccumulation of Hg in fish was only relying on the passive environmental stress of Hg, instead of the active uptake. With the low background of Hg, the passive intake of Hg can be metabolized largely by different fish [5], so that Hg can be only accumulated in low content among fish, which confirmed that the content of Hg in angling fish was even significantly lower than that in market fish (shown in Table 1). The overall low content of Hg in fish degraded the differentiation of fish growth on the accumulation of Hg, so that fish length and weight should be more practical to distinguish metals with high accumulation in fish.

### 3.2. The Contamination of Metals in Angling Fish

**Figure 2:**
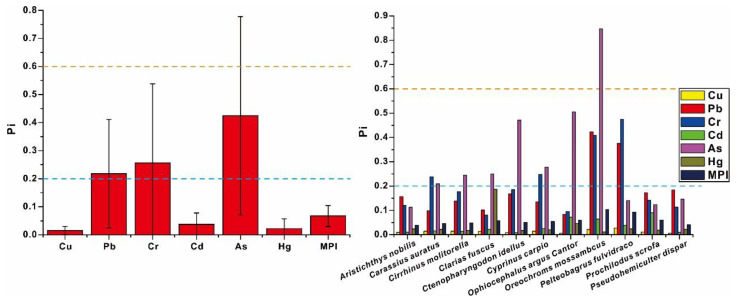
The contamination of metals in angling fish of Guangzhou City. Note: The blue and yellow dotted lines are, respectively, the threshold of low contamination and moderate contamination.

The Pi values of metals were computed to assess metals contamination in angling fish, which was given in Figure 2. The mean values of Pi decreased the order of As > Cr > Pb > Cd > Hg > Cu; among them, the mean Pi of As, Cr, and Pb was found to be beyond 0.2, which suggested these angling fish were commonly minorly contaminated with As, Cr, and Pb. According to the MPI of different fish species, the contamination of metals in different fish species decreased in the order as follows: *Oreochroms mossambcus*, *Pelteobagrus fulvidraco*, *Prochilodus scrofa*, *Ophiocephalus argus Cantor*, *Clarias fuscus*, *Cyprinus carpio*, *Ctenopharyngodon idellus*, *Cirrhinus molitorella*, *Carassius auratus*, *Pseudohemiculter dispar*, and *Aristichthys nobilis*. With the exception of *Pseudohemiculter dispar*, *Aristichthys nobilis,* and *Prochilodus scrofa*, other fish species were commonly contaminated with As, Cr, and Pb, which was confirmed by their Pi that was higher than 0.2. The most contaminated fish were found to be *Oreochroms mossambcus*, which were not only simultaneously contaminated with As, Cr, and Pb, but also the Pi value of As was even beyond 0.8, suggesting a moderate contamination. Therefore, *Oreochroms mossambcus* should be considered as the species that enriches metals easily, which has the potential to be used for the remediation of metal pollution in rivers. On the contrary, the accumulation of metals in *Pseudohemiculter dispar*, *Aristichthys nobilis,* and *Prochilodus scrofa* were relatively low; their Pi of all metals were not beyond 0.2, which suggested the non-pollution of metals on them. Therefore, *Pseudohemiculter dispar*, *Aristichthys nobilis,* and *Prochilodus scrofa* should be treated as the species that does not accumulate metals easily and is safer to consume. The analysis of Monte Carlo was employed in this study to determine the probability of the contamination of As, Cr, and Pb, which is conducive to further recovering metals contamination among angling fish. The results of the analysis of Monte Carlo were given in Figure S2. For Cr and Pb, the Pi values of about 40% angling fish were beyond 0.2, which suggested 40% angling fish were contaminated with Cr and Pb, but most of them had minor contamination, which was confirmed by the extremely high probability of Pi values between 0.2 and 0.6 where 32.2% was for Cr and 31.7% was for Pb, respectively. The moderate contamination of Cr and Pb was not common among angling fish, which was determined by the low probability of Pi values between 0.6 and 1 where 6.9% was for Cr and 3.4% was for Pb, respectively. While the severe contamination of Cr and Pb was extremely low, the probability of Pi values that exceeded 1 was only 2.4% for Cr and 1.4% for Pb, respectively. For As, the probability of a Pi value beyond 0.2 was found to be about 70%, which suggested nearly three quarters of angling fish were contaminated with As. The probability of a Pi value above 0.2 was 42.6%, which was significantly higher than that of Cr and Pb, but all of them are dominantly in 0.2 < Pi < 0.6, which highlighted the widespread minor contamination of metals among angling fish. The probability of 0.6 < Pi < 1 and Pi > 1 was only 15.1% and 9.9%, which expressed the less common moderate and severe contamination among angling fish. Given the widespread mining, metallurgical industry, electroplating, and electronics manufacturing along Pearl River, the relevant contamination of As, Cr, and Pb was confirmed in previous studies [16,17,18,19,33], but their results were not entirely identical with this study. Previous studies indicated the contamination of Cr and Pb among wild fish was found to be more prevalent in Pearl River, while the proportion that was associated to the pollution of As was determined to be relatively small among wild fish in Pearl River [16,17,18,19,33]. However, comparing Cr and Pb, the contamination of As was confirmed to be more prominent in this study. The proportion of As contamination and the contamination degree of As are all significantly higher than that of Cr and Pb in angling fish. More than half of angling fish have moderate As pollution, which probably suggests the difference of metals contamination in Guangzhou City from that in the whole Pearl River Deltas and highlights the severe situation of As pollution in the Guangzhou City to a certain extent. Therefore, the reduction in arsenic emission should be taken into consideration to be a long-term strategy for the ecological restoration of the Guangzhou City, which is of great significance for optimizing fishery environment in Guangzhou City.

### 3.3. The Health Risk Associated to Metals in Fish

The health risks of fish consumption were computed according to children and adults as given in Table 2. The THQ values of metals decreased in the following order: As > Cr > Se > Hg > Zn > Pb > Cu > Cd; among them, the THQ of As was determined to be significantly higher, but it was not beyond 1. Despite the mean THQs of each metal all being down below 1 regardless of adults and children, the TTHQs of adults and children that exceeded 1 still suggested the consumption of these angling fish might pose a substantial threat on the health of the consumer. For the maximal THQ of each metal, only Cr, As, and Hg were found to be higher than 1, which suggested the health risk of angling fish consuming should be largely related to the excessive intake of Cr, As, and Hg. In order to recover the prevalence of the unacceptable risk of angling fish consuming, the analysis of Monte Carlo was applied to calculate the cumulative percentages of THQ for Cr, As, and Hg (Figure S3). The probability of THQ > 1 are relatively low for Cr and Hg, especially for Hg; they are all below 1% regardless of children and adults. For Cr, the probability of THQ > 1 is merely 5.6% for children, but still less than 1% for adults. Despite that the consumption of some fish will also cause exposure to excessive Cr and Hg, their probabilities are still very low, which is inadequate to determine the impact on the overall security of fish consuming. However, for As, the probabilities of THQ > 1 are significantly high for adults and children, which is, respectively, 24.2% and 50.8%. Namely, 24.2% of fish consumption will lead to excessive intake of As by adults, while 50.8% of fish consumption will pose a threat to the health of children, indicating that excessive intake of As should be considered as a critical source of hidden danger in consuming angling fish. Given the prevalent contamination of As among angling fish of Guangzhou City, there is a reason to believe that the contamination of As should be treated as the culprit to reduce the security of angling fish consuming in Guangzhou City.

**Figure 3:**
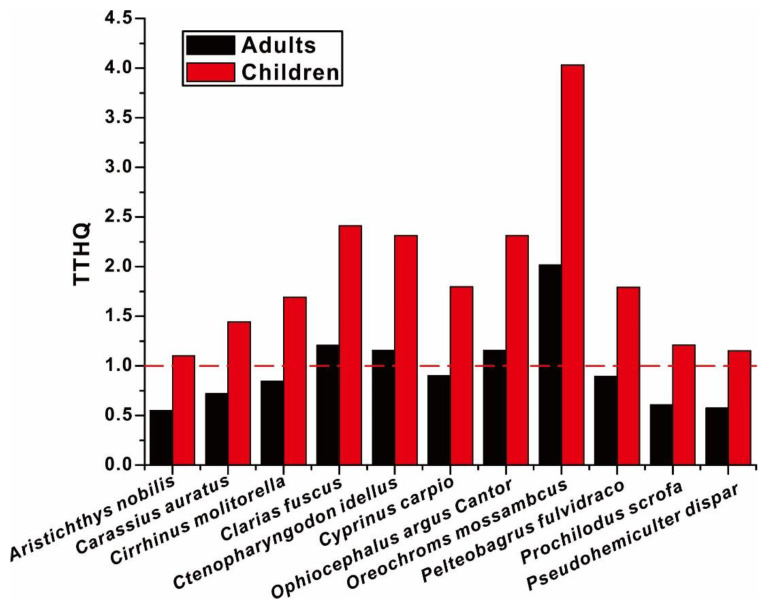
The TTHQ of different fish species for children and adults.

The TTHQ of different fish species is given in Figure 3. For the fish species, the TTHQ decreased in the order as follows: *Oreochroms mossambcus* > *Clarias fuscus* > *Ctenopharyngodon idellus* > *Ophiocephalus argus Cantor* > *Cyprinus carpio* > *Pelteobagrus fulvidraco* > *Cirrhinus molitorella* > *Carassius auratus* > *Prochilodus scrofa* > *Pseudohemiculter dispar* > *Aristichthys nobilis*; among them, the maximal TTHQ was found to be *Oreochroms mossambcus*, which suggested that the consumption of *Oreochroms mossambcus* would have the highest risk to anglers than that of other fish species. In fact, the previous analysis of this study not only confirmed the widespread comtamination of As in *Oreochroms mossambcus* but also disclosed the concurrent contamination of Pb and Cr. Given the overall low THQ of Pb and Cr, the relatively higher TTHQ of *Oreochroms mossambcus* should be tightly related to the high contamination of As among *Oreochroms mossambcus*. For adults, despite the TTHQ of most fish species was down below 1, which suggested an acceptable risk in most fish species for fish consuming, the TTHQ of *Oreochroms mossambcus*, *Clarias fuscus*, *Ctenopharyngodon idellus*, and *Ophiocephalus argus Cantor* that exceeded 1 should be not overlooked, which suggested an unacceptable health risk for consuming these fish species. Therefore, the restriction of fish consumption should be only confined to some fish species, instead of all angling fish. For children, the THQs of all fish species were higher than 1, which suggested no matter what fish species they consumed, they were inevitably exposed to an enormous health risk. Therefore, the angling fish should be not considered to be a regular kind of fish for children to consume.

### 3.4. The Determination of the Screening Size for Acceptable Risk Angling Fish

**Figure 4:**
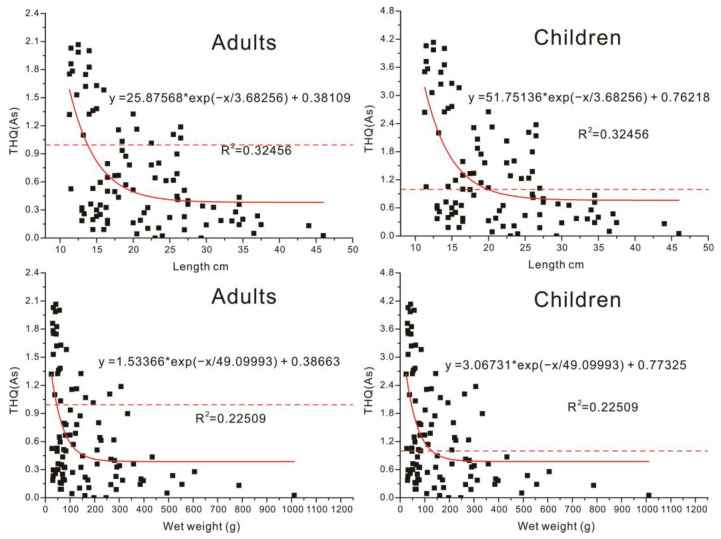
The analysis of regression between length and weight and THQ (As).

**Figure 5:**
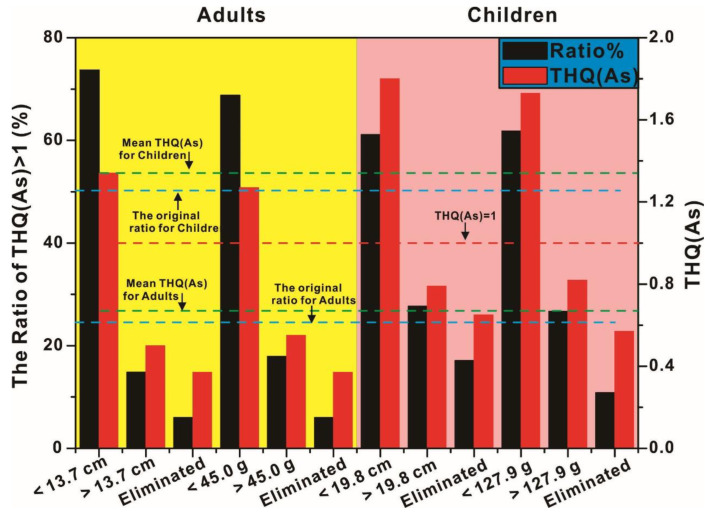
The variation of THQ in screened fish and unscreened fish. Note: Eliminated means *Oreochroms mossambcus* was eliminated from size screened fish.

The analysis of regression was employed in this study to determine the screening size for an acceptable risk in angling fish. The THQ values of As were adopted in this study, due to their maximal influences on the security of fish consuming than that of other metals. As the direct indicators of fish size, the length and weight of fish were used to establish the correlation between THQ and screen size, respectively. The scatterplot is given in Figure 4. The negative correlation between THQ and fish length and weight came out to be significant, as shown in Figure 4, which suggested the high THQ values were primarily centralized in the fish with light weight and short length. With an increase in fish length and weight, most THQ values fall back below 1, which suggests the consumption of most big fish should be acceptable. Therefore, the size screening should be considered to be potential to reverse the high risk of angling fish consuming. All the scatterplots of THQ and fish length and weight are generally consistent with the exponential relationship, so that the exponential regression was selected in this study to determine the suitable length and weight for the acceptable risk in fish screening. For adults, THQ = 1 was incorporated into the regression equation to calculate the corresponding length and weight of fish, which was, respectively. 13.7 cm and 45.0 g. When the length and weight of angling fish were below 13.7 cm and 45.0 g, the THQ values of angling fish were more likely to be higher than 1. Conversely, the THQ values of angling fish were more likely to be within 1, once the length and weight of angling fish were beyond 13.7 cm and 45.0 g. All of this suggested high security in consuming the fish with the length and weight exceeding 13.7 cm and 45.0 g, which also expressed the following statistics. After screening the fish with length and weight exceeding 13.7 cm and 45.0 g, the THQ values of these fish halved to significantly down below 1 (Figure 5), which suggested the consumption of the screened fish should be considered to be overall acceptable. However, the 14.81% and 17.86% THQ values of screened fish can still be found exceeding 1, which suggested that the health risk of angling fish consuming cannot be completely eliminated by only relying on size screening. For children, the corresponding length and weight were calculated to be, respectively, 19.8 cm and 127.9 g via incorporating THQ = 1 into the regression equation. Based on previous studies, the tolerance of metals for children was considered to be low [34,35], so that they could only consume the bigger fish with lower content of metals to offset the health risk of metals, which was confirmed by the significantly higher calculated fish length and weight than that of adults. The THQ of angling fish will tend to be below 1, only if their length and weight are beyond 19.8 cm and 127.9 g, which manifests that the acceptable fish for children to consume needs to exceed the length and weight of 19.8 cm and 127.9 g. Despite the THQ levels of the length and weight of the screened fish being sharply down below to 0.8 and the ratio of THQ > 1 significantly halved too, the THQ of about a quarter of the screened fish was still found to be higher than 1, which suggested the consumption of the screened fish may also have a certain probability to impact the health of Children.

Although fish length and weight screening can be used to degrade the health risk of angling fish to an acceptable range of THQ < 1 for both adults and children, the remaining ratio of THQ > 1 among screened fish still makes it necessary to remain alert to the consumption of angling fish for adults and children. In order to further decrease the remaining ratio of THQ > 1 among screened fish, *Oreochroms mossambcus* was noticed in this study due to their prevalent metals contamination, which may have a vital role in pushing up the health risk of screened fish. For adults, the THQ of 80% *Oreochroms mossambcus* in screened fish were found to be higher than 1, regardless of screening by fish length and weight, while the THQ of all *Oreochroms mossambcus* in screened fish were determined to be beyond 1 for children, no matter the angling fish screened by length or weight. All of these results revealed the ineffectiveness of size screening and excluding *Oreochroms mossambcus* with THQ > 1, which should be blamed on the relatively high contamination of metals in *Oreochroms mossambcus*, especially, the significant bioaccumulation of As. Therefore, it is indispensable to exclude the heavily contaminated *Oreochroms mossambcus* to further drop the health risk of angling fish consuming, even if the angling fish went through the size screening. In this study, after excluding *Oreochroms mossambcus* in the fish screened for size, the THQ of angling fish further dropped down to 0.37, which confirmed the further promotion of the edible security of angling fish. In view of the fact that the ratio of THQ > 1 among screened fish have already dropped to 6% after excluding *Oreochroms mossambcus*, which confirmed the relatively limited impact on the health of adults via angling fish consuming; the consumption of the screened fish should be overall safe for adults with the exclusion of *Oreochroms mossambcus*. For children, the exclusion of *Oreochroms mossambcus* also further decreased the THQ of fish screened for size and further enhanced the acceptability of angling fish consuming, which also expressed the significantly dropped ratio of THQ > 1 to nearly 10%. This relatively low ratio of THQ > 1 among screened fish enables children to consume these angling fish relatively securely. Therefore, the exclusion of the contaminated species should be useful to further degrade not only the values of THQ but also the ratio of THQ > 1, which works for either adults or children.

Generally, the exclusion of the contaminated species could be treated as a supplementary means to strengthen the effects of size screening to further decrease the health risk of angling fish consuming. Although the THQ of adults are all significantly lower than that of children either for the screened fish or unscreened fish, for adults, the THQ of angling fish that went through size screening and contaminated species excluding was merely dropped to 55% of that of unscreened fish. However, for children, a greater decrease in THQ of screened fish was found in this study, the THQ of which was only 43% of that of the unscreened fish. More drops in THQ for children highlighted the superiority of fish screening on mitigating the health risk of fish consuming for children. In view of that, THQ for children should be more likely to be beyond 1 [21], but they may still fall back to 1 by screening fish; therefore, size screening should be of greater practicability in decreasing the health risk of angling fish consuming for children.

## 4. Conclusions

The concentration of Cr and As were relatively high and beyond MRL in some fish. The mean Pi of As, Cr, and Pb were beyond 0.2, which suggested widespread minor contamination among fish. The higher MPI suggested *Oreochroms mossambcus* was the most contaminated. The THQ of Cr, As, and Hg were relatively higher, but the probability of THQ > 1 indicated the health risk should be dominantly from As. The highest TTHQ suggested the highest risk of Oreochroms mossambcus. Regression analysis determined the fish of THQ < 1 should be more likely centralized in size beyond 13.7 cm and 45.0 g for adults and 19.8 cm and 127.9 g for children and save fish of these sizes as screened fish. The significantly reduction in THQ among these screened fish confirmed their effect on degrading the health risk of metals, particularly when THQ for children falls back to within 1. The commonly contaminated *Oreochroms mossambcus* were further excluded from screened fish and the further decrease in THQ confirmed that the exclusion of contaminated species could improve the effect of size screening. The decline in THQ suggested the superiority of fish size and species screening on mitigating the health risk.

## Figures and Tables

**Table 1:** Concentrations of metals in fish of this study and the available literature.

	Cu	Pb	Zn	Cr	Cd	As	Hg	Se
Unit: mg/kg								
Guangzhou City, China	0.225–6.051	0.012–0.412	5.209–42.662	0.071–3.682	0–0.021	0.007–0.624	0.002–0.105	0.199–1.648
	0.771	0.109	16.723	0.512	0.004	0.212	0.007	0.771
Pearl River Delta [25]	1.26	0.943	22.3	0.44	0.038	0.535	-	-
Yangtze River [26]	1.02	0.117	12.193	0.42	0.062	0.095	0.043	-
Yellow River Delta [15]	1.03	0.21	12.1	0.32	0.02	-	-	-
Marketed Freshwater Fish, China [6]	1.119	0.033	-	0.131	0.007	0.039	0.039	-
Maximum Residue Limits (MRL) *	50	0.5	-	2	0.1	0.5	0.3	-

* Pb, Cd, and Cr referred to the maximum residue limits of contaminants in food inChina [22], while Cu, Hg, and As were within the safety requirements for non-environmental pollution of aquatic products in China [23].

**Table 2:** The health risk of metals in angling fish of Guangzhou City.

	Cu	Pb	Zn	Cr	Cd	As	Hg	Se	TTHQ
	Adults								
Min–Max	0.006–0.15	0.003–0.102	0.017–0.141	0.023–1.219	0–0.021	0.022–2.066	0.022–1.045	0.04–0.327	0.347–6.225
Mean	0.019	0.027	0.055	0.169	0.004	0.702	0.066	0.153	1.245
	Children								
Min–Max	0.011–0.301	0.006–0.204	0.034–0.283	0.047–2.438	0.001–0.041	0.045–4.132	0.043–2.09	0.079–0.655	0.695–12.45
Mean	0.038	0.054	0.111	0.339	0.008	1.405	0.132	0.306	2.491

## Data Availability

The original contributions presented in the study are included in the article. Further inquiries can be directed to the corresponding author.

## Supplementary Materials

The following supporting information can be downloaded at: https://www.mdpi.com/article/10.3390/toxics11010054/s1, Table S1. Details of the angling fish of Guangzhou City; Table S2. The correlations between metals content in fish and their length and weight; Figure S1. The concentration of metals in different species of angling fish; Figure S2. The cumulative curve of metals contamination in angling fish of Guangzhou City; Figure S3. The cumulative curve of health risk for As, Cr, and Hg in angling fish of Guangzhou City.
