# Supplementary material for: The Size Screening Could Greatly Degrade the Health Risk of Fish Consuming Associated to Metals Pollution—An Investigation of Angling Fish in Guangzhou, China
Source: Toxics. 2023 Jan 4;11(1):54. doi: 10.3390/toxics11010054 (PMC9861124; doi:10.3390/toxics11010054)
Supplement: Supplementary file 1 [file toxics-11-00054-s001.zip › toxics-2073660-supplementary.pdf]

The Size Screening Could Greatly Degrade the Health Risk of Fish Consuming Associated to Metals Pollution—An Investigation of Angling Fish in Guangzhou, China

Xiongyi Miao [1,2], Qian Zhang [3] and Yupei Hao [2,4,*], Hucai Zhang [4,*]

1 School of Geography and Environmental Science & School of Karst Science, Guizhou Normal University, Guiyang 550001, China
2 Key Laboratory of Karst Dynamics, MNR&GZAR, Institute of Karst Geology, CAGS, Guilin 541004, China
3 Department of CPC Organization and Human Resource, The First Affiliated Hospital of Guangxi Medical University, Naning 530021, China
4 Institute for Ecological Research and Pollution Control of Plateau Lakes, School of Ecology and Environmental Science, Yunnan University, Kunming 650500, China
* Correspondence: yphao66@126.com (Y.H.); zhanghc@ynu.edu.cn (H.Z.)

Table S1. Details of the angling fish of Guangzhou City.

Location	Species	Num	Length cm	Weigh g	Habitat	Feeding habit
Guangzhou City	*Prochilodus scrofa*	8	23-27	176.6-287.9	Demersal	Omnivore
	Ctenopharyngodon idellus	3	18.2-22.5	90.1-127.1	Demersal	Herbivore
	Ophiocephalus argus Cantor	8	26.5-34.5	163.5-434.3	Demersal	Carnivore
	Pelteobagrus fulvidraco	3	12.1-15.2	24.7-31.6	Demersal	Omnivore
	Carassius auratus	11	11.5-23	223.8	Demersal	Omnivore
	Pseudohemiculter dispar	10	13-20	19-60.4	Pelagic	Omnivore
	Cyprinus carpio	30	13-26.5	30.1-606.1	Demersal	Omnivore
	Cirrhinus molitorella	18	16.5-46	56.7-1087.6	Demersal	Omnivore
	Oreochroms mossambcus	35	11.3-26	31.3-334.3	Demersal	Omnivore
	Clarias fuscus	5	22-30	102.6-265.8	Demersal	Omnivore
	Aristichthys nobilis	6	21.5-44	110.7-786.5	Pelagic	Omnivore

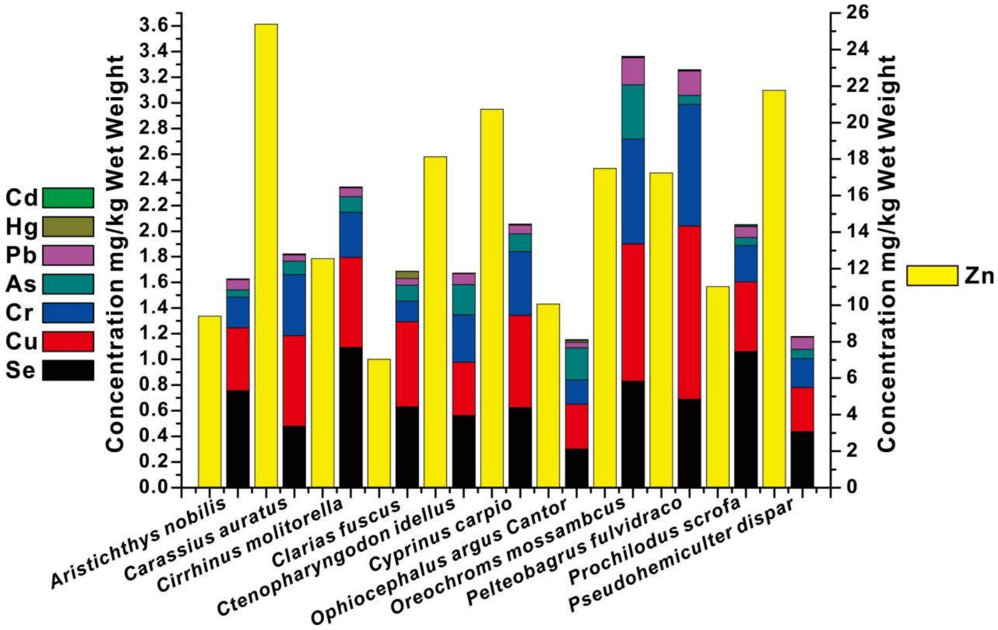

Figure S1. The concentration of metals in different species of angling fish

Table S2. The correlations between metals content in fish and their length and weight.

	Cu	Pb	Zn	Cr	Cd	As	Hg	Se
Weight	**-0.297****	**-0.372****	**-0.435****	**-0.306****	**-0.302****	**-0.402****	0.122	**0.323****
Length	**-0.380****	**-0.502****	**-0.533****	**-0.385****	**-0.343****	**-0.515****	0.194	**0.288****

*. Correlation is significant at the 0.05 level (2-tailed).

**. Correlation is significant at the 0.01 level (2-tailed).

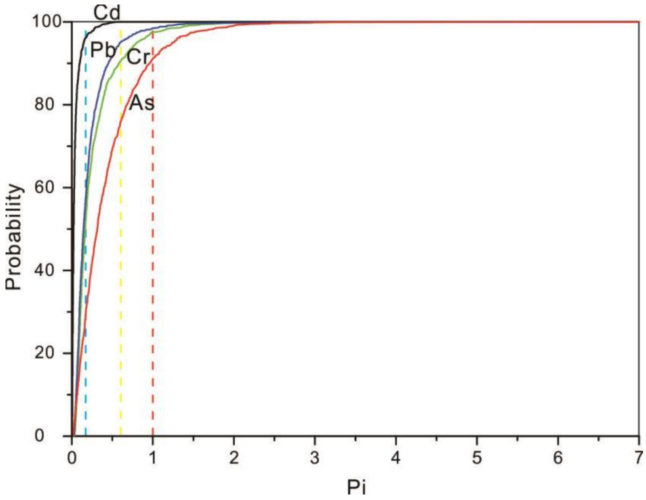

Figure S2. The cumulative curve of metals contamination in angling fish of Guangzhou City

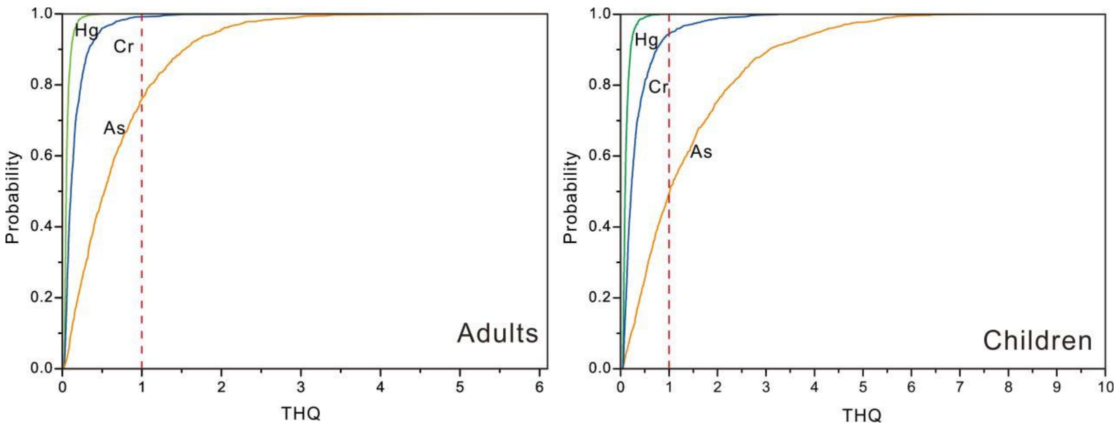

Figure S3. The cumulative curve of health risk for As, Cr and Hg in angling fish of Guangzhou City
